# Activation of brain indoleamine 2,3-dioxygenase contributes to epilepsy-associated depressive-like behavior in rats with chronic temporal lobe epilepsy

**DOI:** 10.1186/1742-2094-11-41

**Published:** 2014-03-04

**Authors:** Wei Xie, Lun Cai, Yunhong Yu, Liang Gao, Limin Xiao, Qianchao He, Zhijun Ren, Yuanzheng Liu

**Affiliations:** 1 Department of Traditional Chinese Medicine, Nanfang Hospital, Southern Medical University, Guangzhou, People’s Republic of China; 2 School of Traditional Chinese Medicine, Southern Medical University, Guangzhou, People’s Republic of China; 3 Southern Medical University, Guangzhou, People’s Republic of China; 4 Department of Encephalopathy, The first affiliated hosipital of Guangxi University of Chinese Medicine, Guangxi University of Chinese Medicine, Nanning, People’s Republic of China; 5 Department of Traditional Chinese Medicine, Guangdong General Hospital, Guangdong Academy of Medical Sciences, Guangdong Geriatric Institute, Guang Zhou 510080, China

**Keywords:** 1-methyltryptophan, Minocycline, Kynurenine, Tryptophan, Serotonin, Interleukin-1β, Interleukin-6, Taste preference test, Forced swim test, Epilepsy, Depression

## Abstract

**Background:**

Depression has most often been diagnosed in patients with temporal lobe epilepsy (TLE), but the mechanism underlying this association remains unclear. In this study, we report that indoleamine 2,3-dioxygenase 1 (IDO1), a rate-limiting enzyme in tryptophan metabolism, plays a key role in epilepsy-associated depressive-like behavior.

**Methods:**

Rats which develop chronic epilepsy following pilocarpine status epilepticus exhibited a set of interictal disorders consistent with depressive-like behavior. Changes of depressive behavior were examined by taste preference test and forced swim test; brain IL-1β, IL-6 and IDO1 expression were quantified using real-time reverse transcriptase PCR; brain kynurenine/tryptophan and serotonin/tryptophan ratios were analyzed by liquid chromatography-mass spectrometry. Oral gavage of minocycline or subcutaneous injection of 1-methyltryptophan (1-MT) were used to inhibite IDO1 expression.

**Results:**

We observed the induction of IL-1β and IL-6 expression in rats with chronic TLE, which further induced the upregulation of IDO1 expression in the hippocampus. The upregulation of IDO1 subsequently increased the kynurenine/tryptophan ratio and decreased the serotonin/tryptophan ratio in the hippocampus, which contributed to epilepsy-associated depressive-like behavior. The blockade of IDO1 activation prevented the development of depressive-like behavior but failed to influence spontaneous seizures. This effect was achieved either indirectly, through the anti-inflammatory tetracycline derivative minocycline, or directly, through the IDO antagonist 1-MT, which normalizes kynurenine/tryptophan and serotonin/tryptophan ratios.

**Conclusion:**

Brain IDO1 activity plays a key role in epileptic rats with epilepsy-associated depressive-like behavior.

## Introduction

Depression represents one of the most common comorbidities of temporal lobe epilepsy (TLE) and has a profoundly negative impact on the quality of life of TLE patients [1]. However, the causes and mechanisms of depression in TLE remain poorly understood. It has been reported that IL-6 mRNA is elevated in the brain of epileptic rats [2,3] and IL-1β expression is also elevated in the brains [4-6] of chronic epileptic animals and children with mesial TLE [6]. Proinflammatory cytokines, such as IL-1β, IL-6 and INF-γ, stimulate indoleamine 2,3-dioxygenase 1 (IDO1) in the brain [7-9]. IDO1 is a rate-limiting enzyme in tryptophan (TRY) metabolism. IDO1 activity has been associated with decreased serotonin (5-HT) content and increased kynurenine (KYN) content and neuroplastic changes through the effect of KYN derivatives, such as quinolinic acid (QUIN), on glutamate receptors, which are all associated with depression [10,11]. IDO1 activation plays a key role in the development of depressive-like behavior. It has been suggested that chronic epilepsy induces the expression of proinflammatory cytokines which, in turn, induce IDO1 expression in the brain. The activation of IDO1 could alter brain TRY metabolism, which contributes to epilepsy-associated depressive-like behavior in rats with chronic TLE.

It has been reported that rats that develop chronic epilepsy following pilocarpine status epilepticus (SE) exhibited a set of interictal disorders consistent with depression [1,12,13]. This model has been further validated as a model of comorbidity between chronic TLE and depression [13]. We examined this hypothesis using a pilocarpine-induced model of chronic TLE in rats.

## Materials and methods

### Animals and treatments

#### Animals

The experiments were performed using 45- to 50-day-old male Wistar rats (Southern Medical University, Guangzhou, People’s Republic of China). The animals were housed and handled in strict accordance with the guidelines of the institutional and national Committees of Animal Use and Protection. Rats were housed in a temperature- (23 to 25°C) and humidity- (45 to 55%) controlled environment with a 12/12-hour modified dark–light cycle (light on 7.00 am to 7.00 pm). The protocol was approved through the Committee on the Ethics of Animal Experiments of the Southern Medical University (Permit Number: SCXK 2013–021).

**Figure 1:**
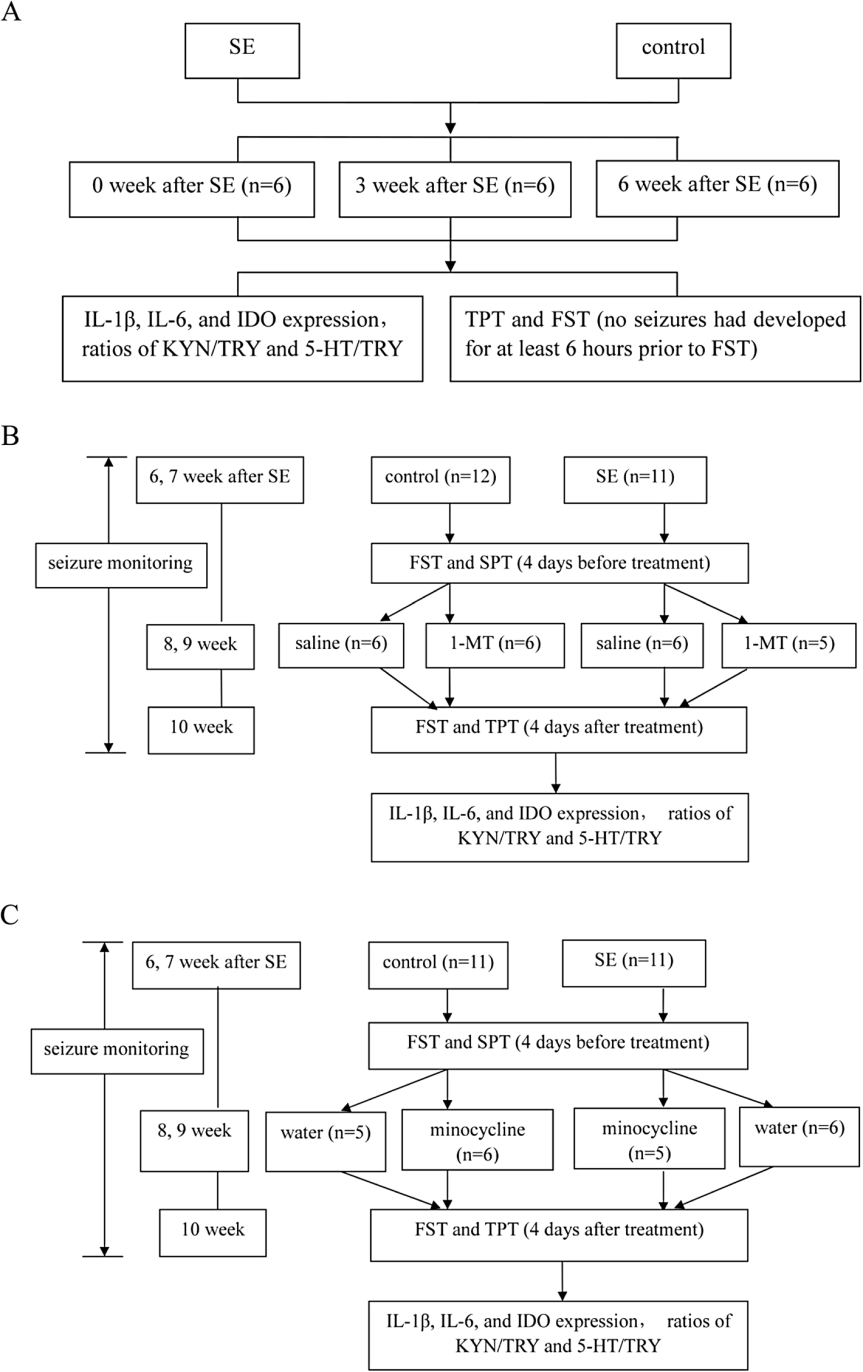
**Study designs. (A)** Study design 1: chronic temporal lobe epilepsy induces depressive behavior. **(B)** Study design 2: 1-methyltryptophan inhibits indoleamine 2,3-dioxygenase 1 directly. **(C)** Study design 3: minocycline inhibits indoleamine 2,3-dioxygenase 1 indirectly.1-MT, 1-methyltryptophan; 5-HT, serotonin; FST, forced swim test; IDO, indoleamine 2,3-dioxygenase; KYN, kynurenine; SE, status epilepticus; TPT, taste preference test; TRY, tryptophan.

Study design is outlined in Figure 1.

### Status epilepticus

SE was induced as previously described [1,12,13]. Briefly, the rats received an intraperitoneal injection of LiCl (130 mg/kg, #310468, Sigma, Buchs, Switzerland). The next day, the animals received a subcutaneous injection of pilocarpine hydrochloride (40 mg/kg, #P0472, Sigma). SE was characterized by continuous limbic seizures which started 10 to 15 minutes after pilocarpine injection. The severity of seizures was evaluated using the Racine [14] scale: (i) motor arrest and twitching vibrissae; (ii) chewing, head bobbing; (iii) forelimb clonus; (iv) forelimb clonus and rearing; and (v) rearing and falling. Rats exhibiting continuous generalized clonic-tonic seizures (which corresponded to stages 4 to 5 on the Racine scale), lasting at least 2 hours, were used for further studies (of 73 pilocarpine-treated rats, 66 met the requirements). After 3 and 8 hours of seizure onset, the rats were injected intraperitoneally with diazepam (5 mg/kg) and phenytoin (50 mg/kg) to alleviate further seizures and increase survival. Control animals received injections of LiCl, diazepam, phenytoin, and saline in lieu of pilocarpine.

### Monitoring spontaneous seizures

To select the proper time points for the forced swim test (FST) and evaluate the effects of treatment on seizure frequency in chronic TLE-induced depression, the behavior of the rats was continuously recorded at 3 and 6 weeks after SE using a digital camera with night vision that could record seizures in the dark (Figure 1A). For experiments using 1-MT (Figure 1B) or minocycline (Figure 1C) to inhibit IDO expression from 6 weeks after SE until the end of the experiment, the behavior of the rats was continuously recorded. Focal seizures (motor arrest, facial twitches and mastication), and generalized clonic or clonic-tonic seizures (all body clonus, rearing or rearing and falling) [1] were analyzed offline. The spontaneous seizure counts were recorded for two consecutive 2-week periods: the first recording was obtained immediately preceding 1-MT/saline or minocycline/distilled water treatment, and the second recording was obtained during the 1-MT/saline or minocycline/distilled water treatment. To analyze the data obtained from post-SE rats treated with 1-MT/saline or minocycline/distilled water, we compared the seizure counts before treatment with those obtained during treatment.

### Behavioral testing

We evaluated depressive behavior using two consecutive tests: taste preference test (TPT) to examine a behavioral correlate of anhedonia (that is, inability to experience pleasure) [1,12,13,15], and FST to study the ability to adapt active strategy in an inescapable stressful situation [1,12,13,16,17]. To examine chronic TLE-induced depressive behavior, we performed FST and TPT at 0, 3 and 6 weeks after SE. For experiments using 1-MT or minocycline to inhibit IDO expression, TPT and FST were performed at 4 days before and after treatment. To avoid the potential immediate effects of seizures, FST was only performed when no seizures had developed for at least 6 hours prior to the test (determined by reviewing video recordings).

TPT was performed using the saccharin solution consumption test as previously described [1,12,13]. Briefly, the animals had free access to a standard rodent diet. On the first day of the experiment (habituation), each cage was supplied with two water bottles containing 250 ml of water. The next day (test), one of the bottles was replaced with 0.1% saccharin (#109185, Sigma) diluted in tap water. The test was initiated at 6.00 pm and ran for 24 hours. TPT was expressed as a percentage of the volume of saccharin solution intake relative to the total water intake (saccharin plus regular water) over 24 hours. The loss of preference for saccharin is indicative of anhedonia [1,12,13,15].

Subsequently, we employed a modified version of the FST in a single 5-minute trial. This modification of the classic test was shown to be relevant for examining a depressive-like state, with the increased immobility time indicating the state of despair [1,12,13,16,17]. Between 4.00 pm and 6.00 pm, the rat was placed for 5 minutes in a glass container (60 cm in height and 30 cm in width) filled with tap water to a height of 45 cm and maintained at 22 to 25°C. The swimming behavior was videotaped and analyzed offline by an investigator blinded to treatment, and the total immobility time was calculated.

### Minocycline and 1-methyltryptophan treatments

Minocycline (#M9511, Sigma) was administered at 120 mg/kg in distilled water through gavage in a dose volume of 10 ml/kg, twice daily, at 12-hour intervals. Previous studies have demonstrated minocycline effectively attenuated IL-1β expression in the brain [8,18].

1-MT (#860646, Sigma) was administered through subcutaneous injection at 50 mg/kg in a dose volume of 5 ml/kg. The injections were administered twice daily at 12-hour intervals, in accordance with the methods of Professor Keith W Kelley, who had administered 50 mg/kg of 1-MT to mice twice a day, and the effect was equal to that observed in studies using 5 mg/day pellets [8]. We prepared 1-MT using 0.1 M NaOH and adjusted the pH to 9.0 using 1 M HCl.

### Real-time reverse transcriptase PCR

Total RNA was extracted from the brain samples using TRIzol reagent (TaKaRa Bio, Dalian, China). The reverse transcriptase reactions were performed on a Stratagene Robocycler Gradient 96 Thermal Cycler (Stratagene, California, USA) using a reverse transcriptase kit (TaKaRa Bio, Dalian, China) according to the manufacturer’s instructions. The single-stranded cDNA was amplified through comparative quantitative real-time reverse transcriptase PCR using a SYBR green Master Mix kit (Thermo Scientific, California, USA; Cat. No. #K0251) on an Mx3005 (Stratagene). The following primers were used: β-actin, (forward) 5′-GCA GGA GTA CGA TGA GTC CG-3′ and (reverse) 5′-ACG CAG CTC AGT AAC AGT CC-3′; IDO1, (forward) 5′-AGC ACT GGA GAA GGC ACT GT-3′ and (reverse) 5′-ACG TGG AAA AAG GTG TCT GG-3′; IL-1β, (forward) 5′-AAA TGC CTC GTG CTG TCT GAC C-3′ and (reverse) 5′-GGT GGG TGT GCC GTC TTT CAT C-3′; and IL-6, (forward) 5′-AGC CCA CCA GGA ACG AAA G-3′ and (reverse) 5′-GGA AGG CAG TGG CTG TCA A-3′. The mRNA expression levels of the target genes were normalized to those of β-actin.

### Liquid chromatography-mass spectrometry

#### Chemicals

Serotonin hydrochloride (#H9523), L-tryptophan (#PHR1176), L-kynurenine (#K8625), and high-performance liquid chromatography-grade methanol were purchased from Sigma. Ultrapure water was generated using a Milli-Q Gradient water purification system (Millipore, Molsheim, France).

#### Apparatus

Liquid chromatography-mass spectrometry (LC-MS) comprised a Prominence 20A series UFLC System (Shimadzu, Kyoto, Japan) and an API 4000 Qtrap MS System (ABSciex, Massachusetts, USA). The analysis was performed using a 100 × 2.1-mm Restek C18 Aqueous column (Restek, Pennsylvania, USA). The LC-MS detection parameters and mobile phases were prepared as previously described [19].

#### Sample preparation

The brain samples were prepared as previously described [20]. Briefly, the frozen brain was dissected on ice into the different regions (prefrontal cortex, midbrain, hippocampus, and thalamus). The samples were subsequently homogenized in a 10-fold volume of a 0.1 M formic acid solution. The homogenates were centrifuged at 18,000 × g for 20 minute at 4°C. The supernatants were collected and stored at −80°C until chromatographic analysis.

### Statistical analysis

All results were expressed as means ± SEM. The statistical analysis was performed using Statistical Product and Service Solutions (SPSS 19.0, Chicago, USA) software (independent samples *t*-test, paired *t*-test, one-way analysis of variance, Wilcoxon test, Mann–Whitney test or Kruskal-Wallis test).

## Results

### Time-dependent induction of depressive-like behavior in chronic epileptic rats

**Figure 2:**
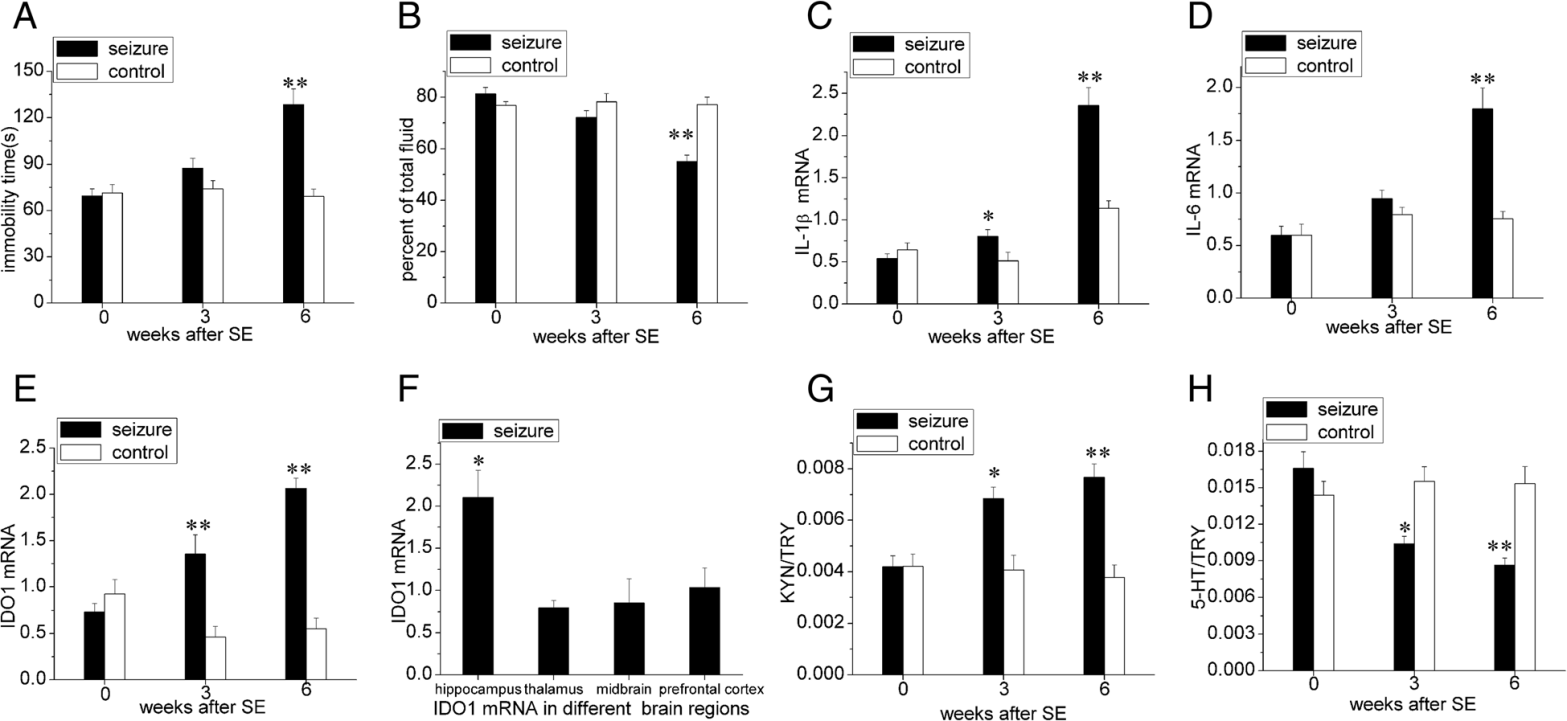
**Chronic temporal lobe epilepsy-induced depressive behaviors, upregulated IL-1β, IL-6 and indoleamine 2,3-dioxygenase expression, and altered tryptophan metabolites. (A)** The duration of immobility during the forced swim test was increased in chronic epileptic rats at 6 weeks after status epilepticus (SE) compared with the controls. **(B)** Saccharin consumption, calculated as a percentage of the total fluid intake over 24 hours, was diminished at 6 weeks after SE. **(C)** IL-1β mRNA levels were gradually increased in chronic epileptic rats at 3 and 6 weeks after SE. **(D)** IL-6 mRNA levels were increased in chronic epileptic rats at 6 weeks after SE. **(E)** Indoleamine 2,3-dioxygenase 1 (IDO1) mRNA levels were also upregulated in chronic epileptic rats at 3 and 6 weeks after SE. **(F)** IDO1 mRNA levels were primarily upregulated in the hippocampus compared with the thalamus, midbrain and prefrontal cortex, and compared with the controls. **(G)** The kynurenine (KYN)/tryptophan (TRY) ratio was elevated and **(H)** the serotonin (5-HT)/TRY ratio was reduced in chronic epileptic rats at 3 and 6 weeks after SE. All the data at 0 weeks are baseline values (naive rats). Statistical significance was determined using independent samples *t*-test and one-way analysis of variance. The data are presented as means ± SEM, n = 5 to 6; *P < 0.05; **P < 0.01.

Chronic TLE induces depressive-like behavior in rats at 6 weeks after SE, but not at 3 weeks after SE, in the FST (Figure 2A, *P* < 0.01) and the TPT (Figure 2B, *P* < 0.01).

### IL-1β and IL-6 expression are induced through spontaneous seizure in the hippocampus of rats with chronic temporal lobe epilepsy

The IL-1β (Figure 2C, *P* < 0.05) mRNA levels were increased at 3 weeks after SE in the seizure group but not in the control group. At 6 weeks after SE, the difference between IL-1β (Figure 2C, *P* < 0.01) and IL-6 (Figure 2D, *P* < 0.01) expression was significant in the hippocampus of rats with coexisting chronic TLE and depressive behavior.

### IDO1 expression is activated through the upregulation of IL-1β and IL-6 expression in the hippocampus of rats with coexisting chronic temporal lobe epilepsy and depressive behavior

**Figure 3:**
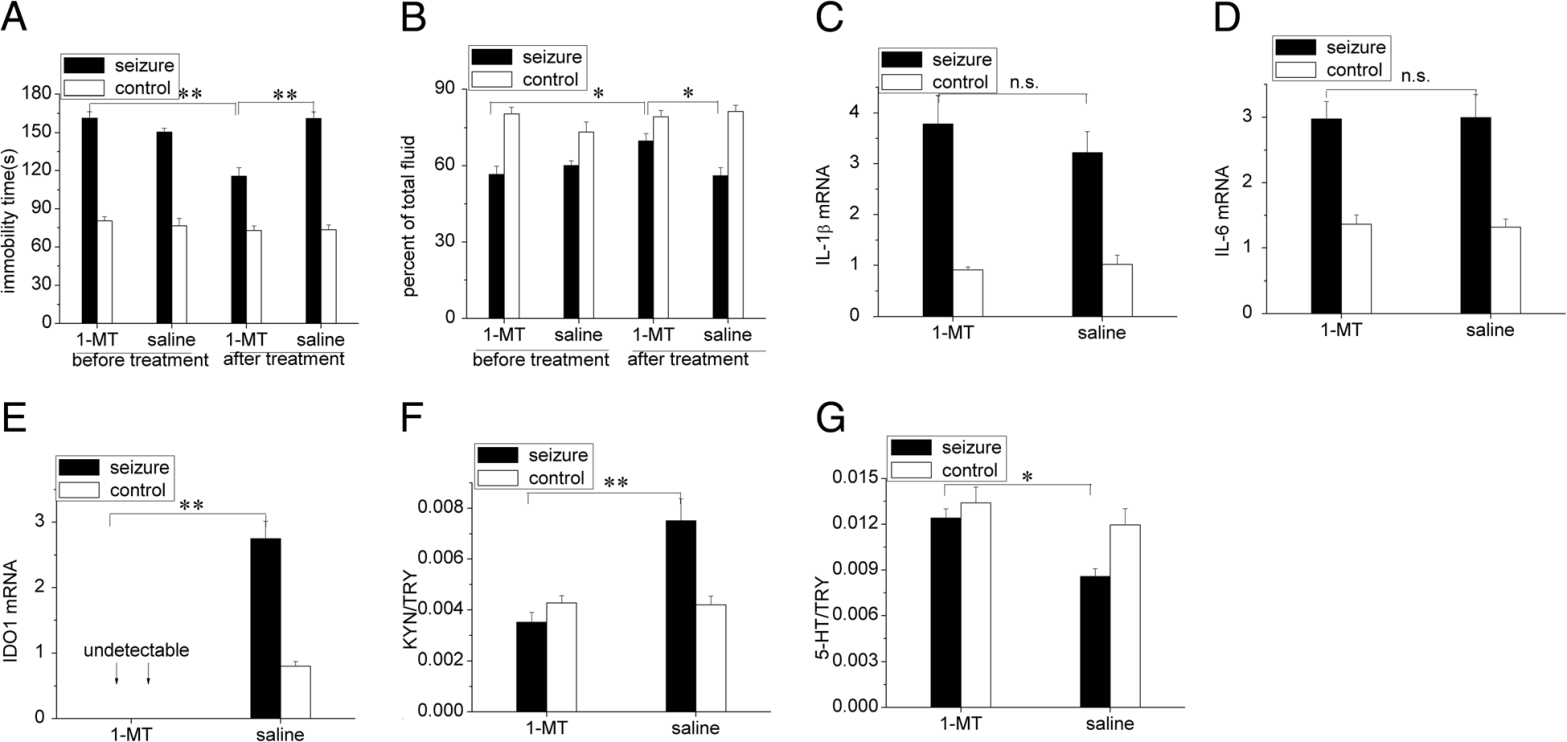
**1-Methyltryptophan blocks chronic temporal lobe epilepsy-induced depressive behaviors, inhibits indoleamine 2,3-dioxygenase expression, and normalizes tryptophan metabolites without reducing IL-1β and IL-6 expression in the hippocampus. (A)** Compared with saline treatment, the immobility time was diminished in the forced swim test (**P < 0.01 versus before treatment by paired *t*-test; **P < 0.01 versus saline treatment by one-way analysis of variance (ANOVA)). **(B)** Saccharin consumption was improved in TPT after 1-methyltryptophan (1-MT) treatment in chronic epileptic rats (*P < 0.05 versus before treatment by paired *t*-test; * *P* < 0.05 versus saline treatment by one-way ANOVA). **(C)** IL-1β and **(D)** IL-6 expression were unaffected by 1-MT treatment but **(E)** indoleamine 2,3-dioxygenase 1 (IDO1) expression was inhibited. **(F)** Kynurenine (KYN)/tryptophan (TRY) and **(G)** serotonin (5-HT)/TRY ratios were normalized in the hippocampus of chronic epileptic rats. Statistical significance was determined using paired *t*-test and one-way ANOVA. The data are presented as means ± SEM, n = 5 to 6; *P < 0.05; **P < 0.01; n.s., not statistically significant (*P* > 0.05).

**Figure 4:**
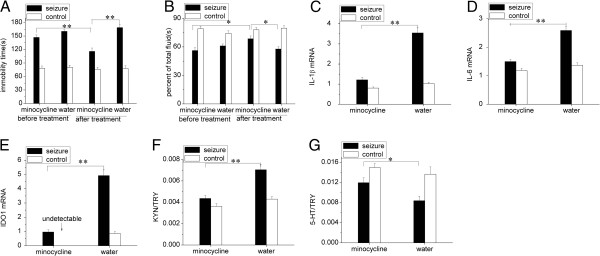
**Minocycline blocks chronic temporal lobe epilepsy-induced depressive behaviors and IL-1β, IL-6 and indoleamine 2,3-dioxygenase expression but normalizes tryptophan metabolites in the hippocampus. (A)** The duration of immobility during the forced swim test was decreased after minocycline treatment for 2 weeks in chronic epileptic rats (**P < 0.01 versus before treatment by paired *t*-test; **P < 0.01 versus distilled water treatment by one-way analysis of variance (ANOVA)). **(B)** Saccharin consumption was also improved in the TPT after minocycline treatment (*P < 0.05 versus before treatment by paired *t*-test; *P < 0.05 versus distilled water treatment by one-way ANOVA). The mRNA levels of **(C)** IL-1β, **(D)** IL-6 and **(E)** indoleamine 2,3-dioxygenase 1 (IDO1) were downregulated in the hippocampus after minocycline treatment. The hippocampal **(F)** kynurenine (KYN)/tryptophan (TRY) and **(G)** serotonin (5-HT)/TRY ratios were normalized. Statistical significance was determined using paired *t*-test and one-way ANOVA. The data are presented as means ± SEM, n = 5 to 6; * *P* < 0.05; ** *P* < 0.01.

IDO1 expression was significantly upregulated at both 3 and 6 weeks after SE (Figure 2E, *P* < 0.01 compared with controls). An examination of the IDO1 expression in different regions showed a significant increase in the hippocampus, compared with the thalamus, midbrain and prefrontal cortex (Figure 2F, *P* < 0.05). These results verified that the hippocampus was a vital region for the upregulation of IDO1 expression. We also observed that, compared with saline treatment, 1-MT specifically inhibited IDO1 expression (Figure 3E, *P* < 0.01) without affecting IL-1β (Figure 3C, *P* > 0.05) and IL-6 (Figure 3D, *P* > 0.05) expression in the post-SE group; compared with distilled water treatment, minocycline inhibited the expression of IDO1 (Figure 4E, *P* < 0.01), IL-1β (Figure 4C, *P* < 0.01) and IL-6 (Figure 4D, *P* < 0.01) in the post-SE group. These results showed that IL-1β and IL-6 induced IDO1 expression.

### Ratios of hippocampal tryptophan metabolites are altered through increased IDO1 enzyme activity

To examine the role of IDO1 enzyme activity in TRY metabolism in both the post-SE and control groups, we first measured TRY, 5-HT, and KYN concentrations in the hippocampus using LC-MS and subsequently determined the ratio of 5-HT or KYN to TRY. There were no baseline differences in the KYN/TRY or 5-HT/TRY ratios between the two groups (Figure 2G and H). However, at 3 and 6 weeks after SE, the KYN/TRP ratio was increased (Figure 2G, *P* < 0.05 at 3 weeks after SE and *P* < 0.01 at 6 weeks after SE) and the 5-HT/TRP ratio was decreased (Figure 2H, *P* < 0.05 at 3 weeks after SE and *P* < 0.01 at 6 weeks after SE) in the post-SE group compared with the control group.

### Both minocycline and 1-methyltryptophan inhibit upregulated IDO1 expression to normalize ratios of hippocampal tryptophan metabolites and block chronic temporal lobe epilepsy-induced depressive-like behavior

To examine whether the inhibition of IDO1 activity influences hippocampal TRY metabolites and depressive behaviors in epileptic rats, we administered the IDO1 inhibitor l-MT (50 mg/kg) or physiological saline twice daily (at 12-hour intervals) for a period of 2 weeks, and the treatments were administered at 8 weeks after SE. The spontaneous seizure counts before or during 1-MT treatment were within the same statistical range in the two chronic TLE groups (Table 1). Treatment with 1-MT, but not saline, lowered the KYN/TRY ratio (Figure 3F, *P* < 0.01), elevated the 5-HT/TRY ratio (Figure 3G, *P* < 0.05) in the hippocampus, reduced the duration of immobility in FST (Figure 3A, *P* < 0.01), and increased saccharin consumption in TPT (Figure 3B, *P* < 0.05).

**Table 1:** Behavioral spontaneous seizure count in post-status epilepticus rats

**Treatment group**	**Before treatment**	**During treatment**
1-MT	2	1
	1	2
	7	4
	30	18
	5	3
Saline	14	17
	7	8
	4	2
	0	0
	13	9
	5	3
Minocycline	5	2
	0	1
	4	0
	27	15
	9	5
Distilled water	9	7
	14	17
	2	0
	17	18
	7	10
	3	2

Before and during minocycline and distilled water treatments, the cumulative spontaneous seizure count in post-status epilepticus rats over two consecutive 2-week periods indicated that no differences were observed between the two periods for each of the groups (*P* > 0.05, both Wilcoxon and Mann–Whitney tests for paired and unpaired comparisons), and across the groups (*P* > 0.05, Kruskal-Wallis test). Similar results were also observed between or across the groups (both *P* > 0.05) with 1-methyltryptophan (1-MT) and saline treatments.

Similar results were obtained after treatment with the cytokine inhibitor minocycline (100 mg/kg) or distilled water twice daily (at 12-hour intervals) for 2 weeks (administered at 8 weeks after SE). No significant difference in the spontaneous seizure count between the two chronic TLE groups (Table 1) was observed, and treatment with minocycline, but not distilled water, also lowered the KYN/TRY ratio (Figure 4F, *P* < 0.01), elevated the 5-HT/TRY ratio (Figure 4G, *P* < 0.01) in the hippocampus, reduced the duration of immobility in FST (Figure 4A, *P* < 0.01, and increased saccharin consumption in TPT (Figure 4B, *P* < 0.05).

Consistent with the fluctuation of IDO1 expression and depressive behavior in the post-SE group, these results suggest that altered ratios of TRY metabolites in the hippocampus indicate increased IDO1 enzyme activity in the hippocampus, which plays a key role in chronic TLE-induced depressive behavior.

### Effect of minocycline and 1-methyltryptophan inhibition on seizures

Before and during minocycline and distilled water treatments, the cumulative spontaneous seizure count in post-SE rats over two consecutive 2-week periods indicated that no differences were observed between the two periods for each of the groups (Table 1, *P* > 0.05) and across the groups (Table 1, *P* > 0.05).

Similar results were also observed between or across the groups (Table 1, both *P* > 0.05) with 1-MT and saline treatments.

## Discussion

Here, we demonstrated that IL-1β, IL-6 and IDO1 expression were selectively upregulated in the hippocampus of rats with chronic TLE and depressive behavior. The blockade of IDO1 activation, either indirectly, with the anti-inflammatory tetracycline derivative minocycline, or directly, with the IDO antagonist 1-MT, prevents the development of depressive-like behavior but failed to affect the occurrence of spontaneous seizures. Both minocycline and 1-MT normalize the KYN/TRY and 5-HT/TRY ratios in the hippocampus of chronic epileptic rats exhibiting depressive behaviors. These results indicated that brain IDO activity plays a critical role in epileptic rats with epilepsy-associated depressive-like behavior.

Epilepsy-associated depression has long been recognized [1,21]. Most studies have focused on the hypothalamo-pituitary-adrenocortical axis, monoaminergic system and various other neurotransmitters/neuromodulators, including GABA and brain-derived neurotrophic factor [22,23]. Despite some progress, the pathogenic mechanisms of epilepsy-associated depression are not completely understood. Although neuroinflammatory pathogenic mechanisms have been identified in rats with coexisting chronic TLE and depression [1], alteration of inflammation in the brain has not been concluded in detail nor completely. To our knowledge, the present study provides the first evidence that experimental chronic TLE is accompanied by interictal IDO upregulation and also verifies alterations in hippocampal inflammation in rats with epilepsy-associated depressive-like behavior.

The data obtained in the present study suggest a novel mechanistic link between epilepsy and depression via IDO1 expression in the hippocampus. The regulation of hippocampal IDO1 expression is likely mediated through upregulated pro-inflammatory cytokines, as the inhibition of IDO1 activity through 1-MT treatment did not prevent the elevation of pro-inflammatory cytokine expression, but the expression of pro-inflammatory cytokines was inhibited with minocycline treatment, which also blocks IDO expression. The data also indicate that pro-inflammatory cytokines are likely induced through chronic TLE. These findings are consistent with previous reports that pro-inflammatory cytokines are elevated in the hippocampus of animal models [1,24-26] and patients [26,27]. In the present study, we also observed that IDO1 was notably upregulated in the hippocampus. These findings are consistent with previous reports that certain brain regions, including the hippocampus, play a critical role in the integration of mood changes and chronic TLE [1,28,29].

KYN and 5-HT are two major TRY metabolites produced through the regulation of metabolic enzymes, including IDO. The results of the present study verified that KYN/TRY and 5-HT/TRY ratios in the hippocampus were regulated through IDO1 activity. Increased IDO1 activity lowers endogenous 5-HT levels; however, this activity also increases KYN derivatives, such as QUIN, a depressogenic glutamate receptor agonist which activates oxidative pathways, causing mitochondrial dysfunctions, and exhibits neuroexcitatory and neurotoxic effects that might lead to neurodegeneration [30]. Moreover, inadequate endogenous 5-HT levels and detrimental increases in the concentration of KYN derivatives lead to depressive symptoms [7,30,31]. Thus, IDO plays a key role in the TRY metabolic pathway, and IDO activation modulates the level of endogenous KYN and 5-HT, leading to epilepsy-associated depressive-like behavior. The proposed mechanism is supported by studies showing that blocks of IDO1 activation either indirectly or directly normalize the increased KYN/TRY ratio and decreased 5-HT/TRY ratio caused by IDO upregulation in the hippocampus. Future studies should address the relationship between IDO expression and other derivatives of TRY metabolism to determine the role of this enzyme in epilepsy-associated depressive-like behavior.

It has been previously reported that pro-inflammatory cytokines might play an important role in epileptogenesis [32-34]. Considering that pro-inflammatory cytokines also play a key role in the pathophysiology of depression [30,35], verified in the present study, these molecules might contribute to the comorbidity between epilepsy and depression. Previous studies have indicated that minocycline might block the epileptogenic process [32,36] or depressive-like behaviors [37,38] through an anti-inflammatory effect. However, in the present study, minocycline administration did not affect spontaneous seizures, as baseline seizure frequencies were relatively low, which likely prohibit the modifying effects of therapeutic interventions. These findings are consistent with those of previous reports [12]. The results of the present study also suggested that the attenuation of depressive symptoms through minocycline administration was not an epiphenomenon of the anticonvulsant effects of this drug.

Although the observed effects of minocycline treatment were different from the 1-MT-meadiated attenuation of depressive symptoms, the anti-epileptic effect of this drug was not verified in the present study. Indeed, IDO1 activation increases KYN derivatives, such as QUIN, a glutamate receptor agonist with neuroexcitatory and neurotoxic effects that induce epilepsy [39,40]. However, this effect has been previously observed in the pathophysiology of depression. Thus, IDO1 activation increases KYN derivatives and likely contributes to the comorbidity between epilepsy and depression. In the present study, 1-MT administration attenuates depressive symptoms through the inhibition of IDO1 activation, which normalizes KYN/TRY and 5-HT/TRY ratios in the hippocampus. However, 1-MT did not affect spontaneous seizures, reflecting either the relatively low baseline seizure frequencies observed in the present study [12] or other mechanisms involved in the described phenomenon. Notably, the experimental protocol used in the present study might not be suitable for the reliable examination of anti-epileptic therapies [1]. Thus, future studies will focus on effects of the TRY pathway and 1-MT treatment on epilepsy.

In conclusion, the results of the present study implicate hippocampal IDO1 activation in epilepsy-associated depressive-like behavior, suggesting that depression in TLE patients could be treated through the regulation of brain inflammation and IDO1 activity. Although the mechanism underlying the relationship between epilepsy and depression is most likely complex and involves other neurotransmitters and neuromodulators [16,18], the results of the present suggest a new strategy for the prevention and reversal of depression in TLE patients through a mechanism involving altered endogenous TRY metabolite ratios via upregulated pro-inflammatory cytokines and IDO expression in certain brain regions.

## Abbreviations

1-MT: 1-methyltryptophan; 5-HT: serotonin; FST: forced swim test; IDO1: indoleamine 2,3-dioxygenase 1; IL: interleukin; INF: interferon; KYN: kynurenine; LC-MS: liquid chromatography-mass spectrometry; PCR: polymerase chain reaction; QUIN: quinolinic acid; SE: status epilepticus; TLE: temporal lobe epilepsy; TPT: taste preference test; TRY: tryptophan.

## Competing interests

The authors declare that they have no competing interests.

## Authors’ contributions

WX contributed to the study design. LC contributed to the study design and participated in the acquisition and analysis of data, the statistical analysis and the manuscript drafting and revision. LG, YY, LX and QH participated in the data analysis and manuscript revision. ZR and YL participated in data acquisition and manuscript drafting and revision. All authors have read and approved the final version of the manuscript.

## Authors’ information

Wei Xie and Lun Cai are co-first authors.
